# Friction Stir Spot Welding of Different Thickness Sheets of Aluminum Alloy AA6082-T6

**DOI:** 10.3390/ma15092971

**Published:** 2022-04-19

**Authors:** Mohamed M. Z. Ahmed, Mohamed M. El-Sayed Seleman, Essam Ahmed, Hagar A. Reyad, Kamel Touileb, Ibrahim Albaijan

**Affiliations:** 1 Mechanical Engineering Department, College of Engineering at Al-Kharj, Prince Sattam Bin Abdulaziz University, Al Kharj 16273, Saudi Arabia; k.touileb@psau.edu.sa (K.T.); i.albaijan@psau.edu.sa (I.A.); 2 Department of Metallurgical and Materials Engineering, Faculty of Petroleum and Mining Engineering, Suez University, Suez 43512, Egypt; mohamed.elnagar@suezuniv.edu.eg (M.M.E.-S.S.); essam.ahmed@suezuniv.edu.eg (E.A.); engineering667@yahoo.com (H.A.R.)

**Keywords:** aluminum alloys, AA6082, friction stir spot welding, tensile shear load, hardness, microstructure

## Abstract

Friction stir spot welding (FSSW) is one of the important variants of the friction stir welding (FSW) process. FSSW has been developed mainly for automotive applications where the different thickness sheets spot welding is essential. In the present work, different thin thickness sheets (1 mm and 2 mm) of AA6082-T6 were welded using FSSW at a constant dwell time of 3 s and different rotation speeds of 400, 600, 800, and 1000 rpm. The FSSW heat input was calculated, and the temperature cycle experience during the FSSW process was recorded. Both starting materials and produced FSSW joints were investigated by macro- and microstructural investigation, a hardness test, and a tensile shear test, and the fractured surfaces were examined using a scanning electron microscope (SEM). The macro examination showed that defect-free spot joints were produced at a wide range of rotation speeds (400–1000 rpm). The microstructural results in terms of grain refining of the stir zone (SZ) of the joints show good support for the mechanical properties of FSSW joints. It was found that the best welding condition was 600 rpm for achieving different thin sheet thicknesses spot joints with the SZ hardness of 95 ± 2 HV0.5 and a tensile shear load of 4300 ± 30 N.

## 1. Introduction

Nowadays, lightweight materials such as aluminum alloys and/or magnesium alloys and their composites are widely used, especially in automotive and aerospace industries, where saving weight is very important [1,2,3]. Spot joining technologies for lightweight materials are laser spot welding, resistance spot welding, and riveting. Disadvantages of these technologies include the consumption of tools during joining, large heat distortion, and poor weld strength joints [4,5,6,7]. Porosity defects for conventional resistance spot welding and laser spot welding cannot be avoided. Moreover, the riveting process increases the weight of joining parts, besides the cost of drilling needed [8,9,10].

New welding technology based on the friction stir welding (FSW) principle has been invented and introduced to the aerospace and most transportation industries with the name of friction stir spot welding (FSSW). The FSSW is recommended as a fast and cheap method to be used intensively for joining main structural components of similar [5,11,12,13,14] and dissimilar materials [15,16]. Thus, this welding technique is considered a strong competitive method for other spot joining technologies [17,18].

The FSSW is a solid-state joining method whereby a rotating tool is plunged into material sheets in a lap joint setup configuration to a certain depth to weld them via frictional heat generated during the friction stir process [18,19]. Thus, the FSSW is a thermo-mechanical process; the tool is surrounded by plasticized stir zone (SZ) material with high temperature and subjected to severe stress, especially for joining. In the FSSW, the solidification defects are avoided compared to the fusion welding techniques, and the joints are performed in a short time [20,21]. It consists of three stages: the plunge stage with the rotating tool under a certain downward force, stirring at a specific holding time, and the drawing out stage. During the plunging stage, the rotating tool faces high resistance from the workpiece. After that, the material is softened as a result of the plastic deformation and the generated frictional heat. During the dwell period, the frictional heat was raised and allowed the material to flow in the weld zone (WZ). In the last stage, the rotating tool is drawn out from the WZ [15,20].

The friction stir spot welded (FSSWed) joint has a characteristic of a keyhole in the middle. The produced FSSWed joint contains various typical zones, such as SZ, thermo-mechanical affected zone (TMAZ), heat affected zone (HAZ), and base material as found in the FSW joints [18]. In order to achieve spot-welded joints, many FSSW processes have been developed, such as pin-less FSSW, refill FSSW, and swing FSSW [2,20].

The AA 6xxx series (Al, Mg, and Si) alloys are frequently used in transportation and aerospace industries due to good corrosion resistance, excellent extrusion performance, and weldability [22,23,24]. Yuan et al. [25] used two different tools with the same shoulder feature and different pin geometries, a conventional pin and off-center pin feature, to produce a similar FSSW lap joint of 1 mm thickness AA6016-T4. The obtained results showed that the two tools exhibited a maximum weld separation load of around 3.3 kN at different rotation speeds of 1500 rpm for the conventional pin and 2500 rpm for the off-center pin. In addition, there is no relation between the microhardness results and separation modes of the welded joints. Gao et al. [18] utilized the GDRX model implemented in DEFORM-3D to forecast grain size during the FSSW of AA6082 and observed that grain size refinement owing to dynamic recrystallization could be predicted. Muhayat et al. [26] studied the effect of the pin diameter (3–7 mm) and dwell time (3–9 s) of FSSW similar thickness AA6082-T6 lap joints and reported an increase in dwell time and that the pin diameters decrease in the hardness of the welded joint. Moreover, the excessive heat input at 7 mm pin diameter and 9 s dwell time resulted in a decrease in tensile shear load. Aydin et al. [5] applied high rotation speeds from 1000 to 2500 rpm at a constant dwell time of 7 s, feeding rate of 50 mm/min, and plunge depth of 5 mm to achieve a similar thickness FSSW lap joint of 3 mm AA6082-T6 sheet. The results showed a drop in the hardness of the welded zone compared to the BM. Aydin et al. [27], in other work, recommended a cylindrical pin profile to FSSW of similar AA6082-T6 lap joint of 3 mm sheet thickness among the different pin profiles of conical, hexagonal, triangular, and cylindrical with two grooves used at the applied welding conditions of a relatively high rotation speed of 1500 rpm at different dwell times from 2 to 11 s. Buffaa et al. [28] FSSWed a similar thickness of AA6082-T6 of 1.5 mm sheet alloy at rotation speeds 900, 1500, and 2000 rpm using a cylindrical pin. The obtained results showed that increasing tool rotation causes a decrease in failure load.

In fact, there is a need in the transportation industry to friction stir spot weld different thin thicknesses AA6XXX. Based on the available literature, there is no try to FSSW of dissimilar thin thickness lap joints of AA6082-T6. Moreover, most researchers focused on using high rotational speeds (900–2500 rpm) to achieve the FSSW [5,27,28]. Thus, this study intended to explore the possibility of FSSW dissimilar thin sheet thickness 1 and 2 mm of AA 6082-T6 alloy at relatively lower rotational speeds of 400, 600, and 800. Moreover, the high rotation speed of 1000 rpm was tried for comparison. The microstructure and mechanical properties in terms of hardness and tensile shear load of the produced joints were examined with the light of applied heat input and the FSSW thermal cycle.

## 2. Methodology

### 2.1. Starting Materials

The starting material was AA6082-T6 aluminum alloy with two different sheet thicknesses of 1 × 1000 × 1000 mm and 2 × 1000 × 1000 mm were supplied by Future Fond Company, Milano, Italy. The two sheets have the same chemical composition as listed in Table 1, and Table 2 summarizes the measured mechanical properties.

### 2.2. Friction Stir Spot Welding Process

**Figure 1:**
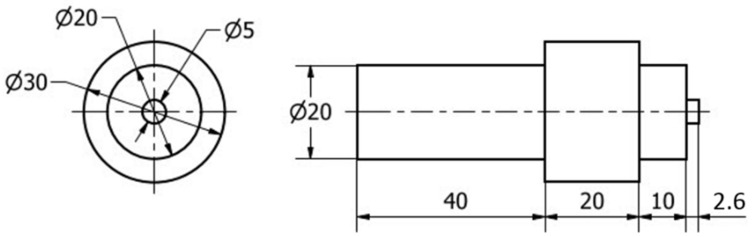
Dimensions of the FSSW tool (all dimensions are in mm).

The FSSW joints were produced using the friction stir welding/processing machine (EG-FSW-M1) [29]. For spot welding purposes, the two sheets were cut to specimens 100 mm in length and 30 mm in width. The specimens were spot welded in lap joints with a 30 mm overlap for the two different sheet thicknesses at a constant dwell time of 3 s and various rotation speeds of 400, 600, 800, and 1000 rpm. The other spot welding parameters in terms of plunge depth, plunge rate, and tilt angle were kept constant at 2.6 mm, 0.1 mm/s, and 0°, respectively. The tool used was made of steel (AISI H13) with dimensions of 20 mm shoulder diameter, 5 mm pin diameter, and 2.6 mm pin length. The tool has a flat shoulder and a cylindrical pin, as shown in Figure 1. The selection of tool geometry and tool material was based on the literature [5,6,30]. The cylindrical pin profile produces axisymmetric temperature distributions and heat transfer phenomena through the welds. The effect of the circular flow velocity due to the cylindrical pin on axisymmetric temperature distributions is small [6,7,31].

**Figure 2:**
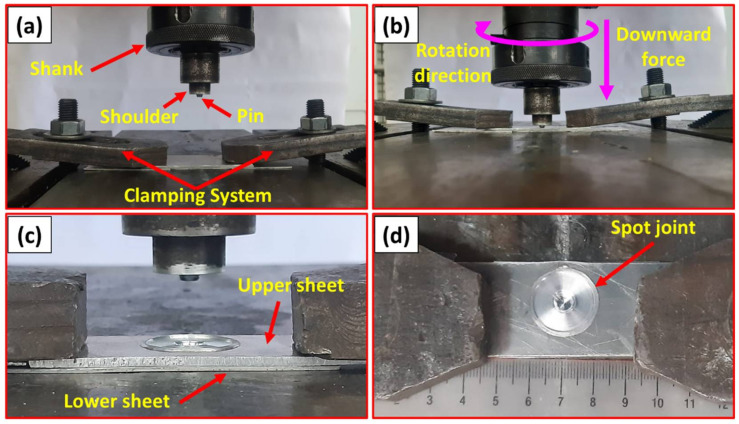
(**a**–**c**) illustrates the steps of the FSSW process of AA6082-T6 lap joints, while (**d**) shows the top view of the produced spot joint.

**Figure 3:**
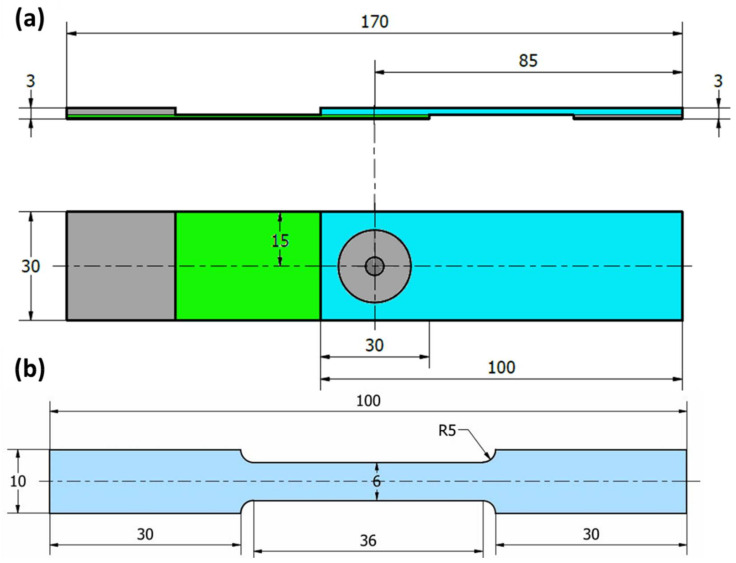
Schematic drawing of (**a**) tensile/shear test samples of FSSW lap joints, (**b**) tensile test sample of AA6082-T6 BM. (All dimensions in mm).

Figure 2 summarizes the FSSW process carried out, which consists of three steps: (1) lap joint fixing with a clamping system (Figure 2a); (2) plunge stage and stirring action with the rotating tool (Figure 2b); (3) the drawing out stage (Figure 2c). Finally, Figure 2d shows the top view of a representative specimen spot-welded dissimilar thickness AA6082-T6 joint. The modern digital multimeter (type-UT61B, Zhejiang, China) was used to measure the temperature during the FSSW at the applied different rotation speeds (400–1000 rpm) using a thermocouple type “K”.

**Figure 4:**
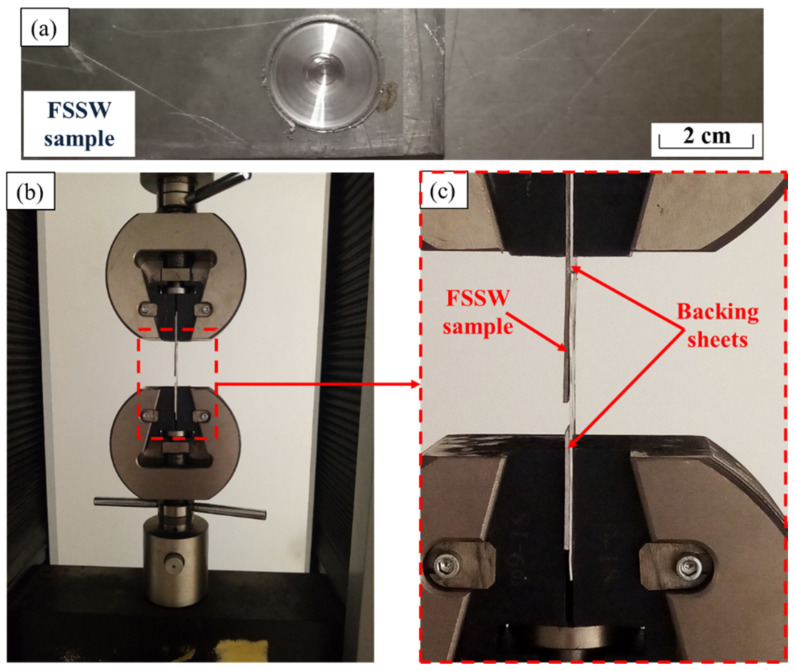
Photo image of (**a**) a dissimilar thickness AA6082-T6 lap joint, (**b**) setup of tensile/shear test of the spot-welded joint, and (**c**) higher magnification of (**b**).

The produced FSSW joints were sectioned for macrostructure evaluation, microstructure investigation, and hardness test. The cross-sectional specimens were ground with SiC papers up to 2400 grit, then polished using polishing cloth in the presence of alumina paste up to a surface finish of 0.05 µm. Chemical etching of the polished samples was performed using two different compositions of Keller’s etcher. The first consists of 25 mL nitric acid (HNO_3_), 25 mL hydrochloric acid (HCl), 25 mL methanol (CH_3_OH), and one drop of hydrofluoric (HF). The other was 2.5 mL HNO_3_, 1.5 mL HCl, 95 mL distilled water, and 1 mL HF. The microstructure investigation was conducted using Olympus optical microscope (OM) (BX41M-LED, Olympus, Tokyo, Japan). The hardness test was performed for obtaining hardness contour maps across the transverse sections of the spot-welded joints. The hardness measurements were carried out using a Vickers hardness tester (model HWDV-75, TTS Unlimited, Osaka, Japan) using an applied load of 5 N with a dwell time of 15 s. The distance between every two indentations was set to 0.75 mm along the cross-section of the welds. The tensile-shear test (the load-carrying capacity) was carried out using a 30-ton universal tensile testing machine (Type-WDW-300D, Guangdong, China) at room temperature. The FSSW joints were tested with a constant loading rate of 0.1 mm/s. The tensile shear test specimens were cut and prepared according to ASTM E 8M-04. Figure 3 illustrates schematic drawings of the tensile-shear test specimen of the FSSWed dissimilar thickness AA6082-T6 joint (Figure 3a) and the tensile test specimen of the BM (Figure 3b). During the tensile-shear test of the joint specimen (Figure 4a), two backing sheets were used to ensure the application of axial load (Figure 4b,c). After tensile testing for the 2 mm thickness BM and the FSSWed fractured joints produced at 400 and 1000 rpm, the fractured surfaces were examined using a scanning electron microscope (SEM-Quanta FEG 250-FEI Company, Hillsboro, OR, USA).

## 3. Results and Discussion

### 3.1. FSSWed Joints Surfaces’ Appearance

**Figure 5:**
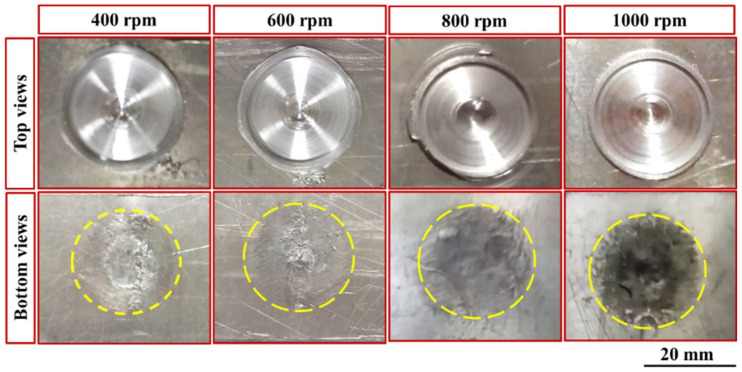
Typical appearances of both sides of dissimilar thin thickness FSSW AA6082-T6 lap joint samples at a constant dwell time of 3 s and different rotation speeds.

Figure 5 shows the top and bottom typical appearances of the FSSW joints produced at a constant dwell time of 3 s and different rotational speeds of 400, 600, 800, and 1000 rpm. From a visual inspection, it can be observed that the applied combination parameters were suitable for producing FSSWed joints between different thickness sheets of AA6082-T6. For the top view of the spot-welded joints (Figure 5), it can be seen that circular indentations due to shoulder projection are observed at the different applied parameters and the extruded material flashed to the sides of the shoulder projection is nearly similar. Based on the experimental and published data from our lab [15,32,33], the processing spot welding parameters were carefully selected to eliminate excessive flash during the FSSW. The bottom view of the FSSW joints shows thermal hot spot areas (the affected areas due to the friction stirring process). These thermal hot spot areas get darker with increasing the rotational speed as a result of increasing the generated heat input during the spot dwell time, as can be seen in Figure 5.

### 3.2. Heat Input Calculation and Temperature Measurement

The heat input generated during the FSSW mainly depends on the tool shoulder profile, pin geometry, rotation speed, friction coefficient, axial downward force, and dwell time [16,33,34]. The thermo-mechanical process during the FSSW converts the produced mechanical energy through tool rotation into heat input in the workpiece. The heat input for the FSSW process (*Q*) can be calculated using Equation (1) [33,35]:

(1) $$Q=\frac{13}{12}\mu \frac{P}{K_{A}}\omega rt\;(J),$$

where $P\left(N\right):\;$applied downward force, $K_{A}:$ the ratio of the contact area of shoulder profile to tool cross-sectional area, ω (rad/s) equals 2π*n n*: the used rotation speed), *t* (s): the applied dwell time during the FSSW process, and $r\;\left(m\right):$ tool tip radius. μ (the friction coefficient between tool and aluminum alloy): equals 0.4 [14].

Where:

(2) $$K_{A}=\frac{shoulder\;radius^{2}-pin\;radius^{2}}{shoulder\;radius^{2}}\;\;0.9375,$$

From Equations (1) and (2), the heat input to produce dissimilar spot lap joints of AA6082-T6 can be calculated using Equation (3).

(3) $$Q=1.1859\times 10^{\left(-3\right)}\times n\times P\times t,$$

**Figure 6:**
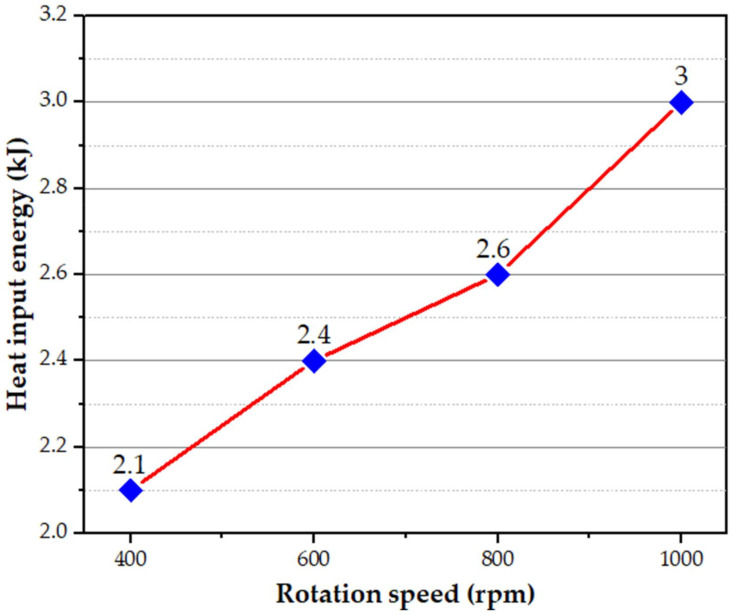
The generated heat input energy during the FSSW against the applied rotational speeds.

Figure 6 illustrates the generated heat input at the applied different rotational speeds from 400 to 1000 rpm during the FSSW of the dissimilar thin thickness AA6082-T6 spot joints. The generated heat input increases with increasing the rotational speed up to 1000 rpm. The rotation speed of 1000 rpm exhibits the highest heat input energy of 3 kJ.

**Figure 7:**
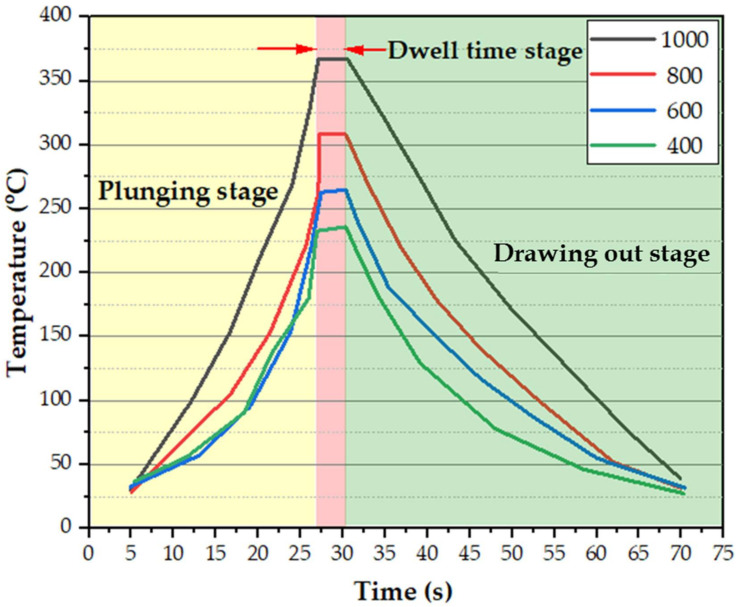
The thermal cycles during the FSSW of the dissimilar thin thickness AA6082-T6 lap joints processed at a constant dwell time of 3 s and the different rotational speeds of 400, 600, 800, and 1000 rpm.

The FSSW thermal cycles of the SZ in terms of the measured working temperature as a function of time at the different rotation speeds are shown in Figure 7. It can be seen here that the thermal cycle gives the same trend at the applied different rotation speeds from 400 to 1000 rpm at a constant dwell time of 3 s, with a difference in the measured peak temperatures at each rotation speed. The FSSW of the dissimilar thin thickness AA6082-T6 lap joints process can be divided into three stages. The first stage records the rising temperature gradually by penetrating the tool pin at a constant feed rate of 0.1 mm/s through the two sheets of the lap joint with direct contact of the tool shoulder with the top surface of the upper sheet at the applied rotation speed. The second stage represents the dwell time needed to achieve the spot joint at nearly temperature stability. The third stage (drawing out stage) represents the end of the welding process and air-cooling of the lap joint with gradually temperature loss. Table 3 lists the peak measured temperature in the SZ during the FSSW process. The temperature increases from 236 ± 4 to 367 ± 3 °C with the increasing rotational speed from 400 to 1000 rpm, respectively.

### 3.3. Transverse Macrographs of the FSSWed Joints

**Figure 8:**
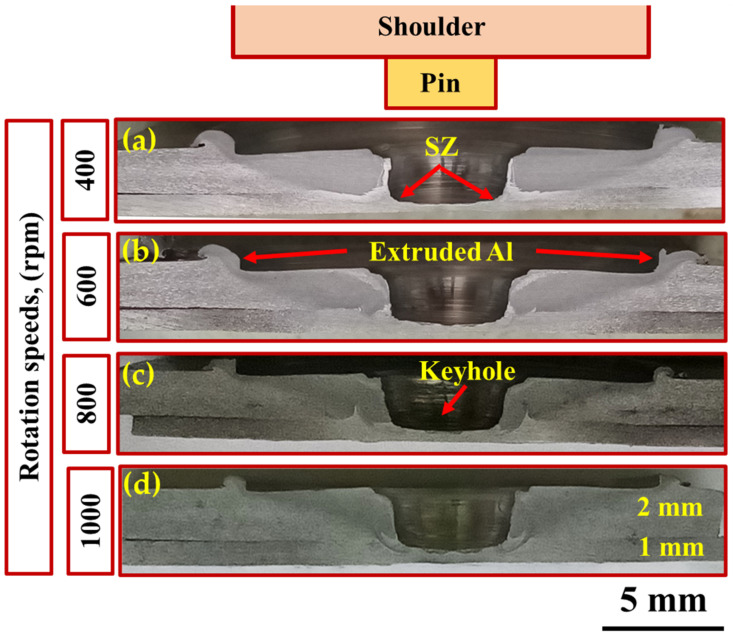
Macrographs of the transverse cross-section of the FSSW lap joints at a constant dwell time of 3 s and rotational speeds of (**a**) 400 rpm, (**b**) 600 rpm, (**c**) 800 rpm, and (**d**) 1000 rpm.

Figure 8 shows the transverse cross-sections macrographs of the FSSWed AA6082-T6 joints. It can be observed that the interface between the overlapped sheets is welded due to plastic deformation and material flow produced by the rotation of the tool pin. In previous studies [15,36], it has been reported that three different regions are established in the spot weld zone. These regions are the flow transition zone under the rotating tool shoulder, the SZ around the tool pin, and the torsion zone under the pin. In fact, the joint efficiency of the FSSW is controlled by various parameters, including welding material, machine parameters (rotational speed, dwell time, and downward force), and tool material and design [1,25,27].

### 3.4. Microstructure Investigation

**Figure 9:**
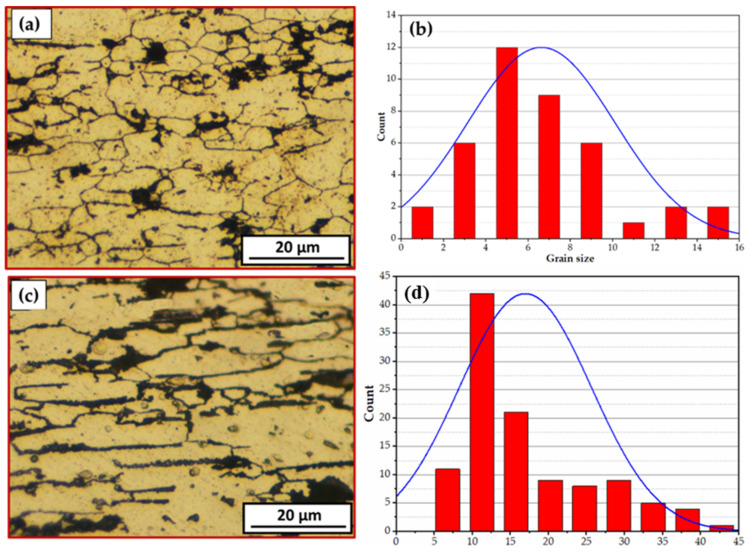
OM images, (**a**) and (**c**) and grain size histograms, (**b**) and (**d**) of AA6082-T6 sheet BMs of 1 mm and 2 mm thickness, respectively.

The microstructures and grain size histograms of the AA 6082-T6 BMs are shown in Figure 9. It can be seen that the BM microstructures show elongated and larger grain structures in the rolling directions, including very fine dispersed Mg_2_Si precipitates and relatively coarse (Fe, Mn)_3_SiAl_l2_ particles, as shown in Figure 9a,c. Typical microstructure features of the AA 6082-T6 BM are reported by Aydin et al. [27]. It can be remarked that AA 6082-T6 sheets of 1 and 2 mm thickness have mean grain size values of 6.63 ± 2 μm and 14.14 ± 1.5 μm, as shown in Figure 9b,d, respectively. The difference in grain size of BMs is related to imposing intense plastic deformation of thickness reduction. More thickness reduction using the rolling process in asymmetric directions causes high internal strain and grain size refining that increases the elongation and the strength [37].

**Figure 10:**
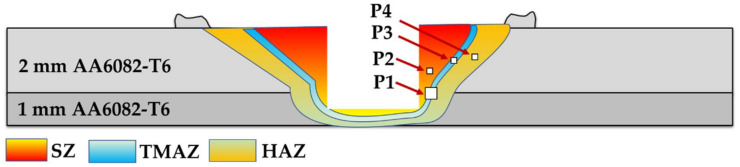
Schematic drawing showing the locations of the selected points (P1–P4) for the microstructure examination of the friction spot weld zone.

**Figure 11:**
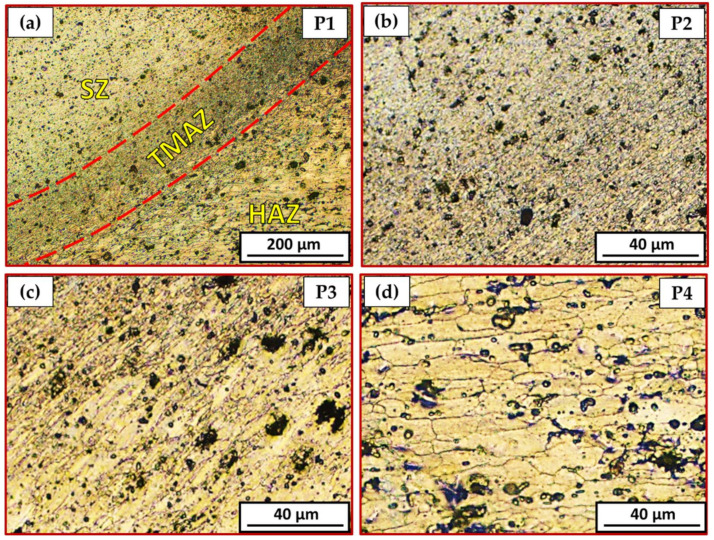
Different microstructure zones of the joint spot welded at 400 rpm where P1 is mixed zone of SZ/TMAZ/HAZ in (**a**), P2 is SZ in (**b**), P3 is TMAZ in (**c**), and P4 is HAZ in (**d**).

**Figure 12:**
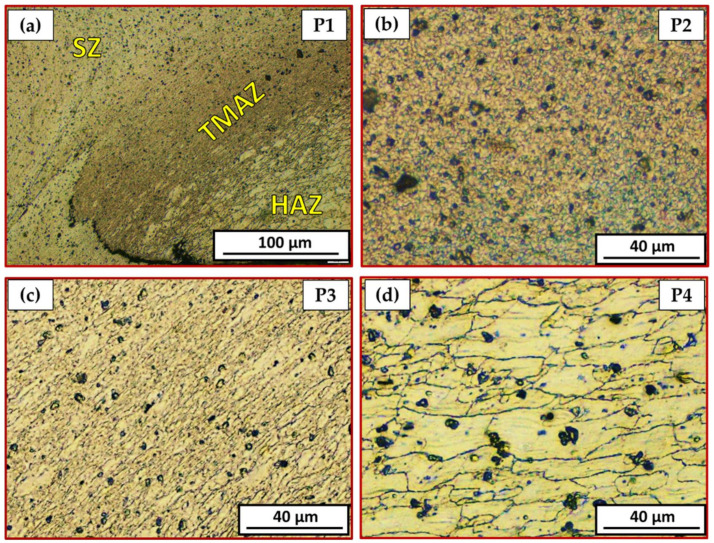
Different microstructure zones of the joint spot welded at 600 rpm where P1 is mixed zone of SZ/TMAZ/HAZ in (**a**), P2 is SZ in (**b**), P3 is TMAZ in (**c**), and P4 is HAZ in (**d**).

**Figure 13:**
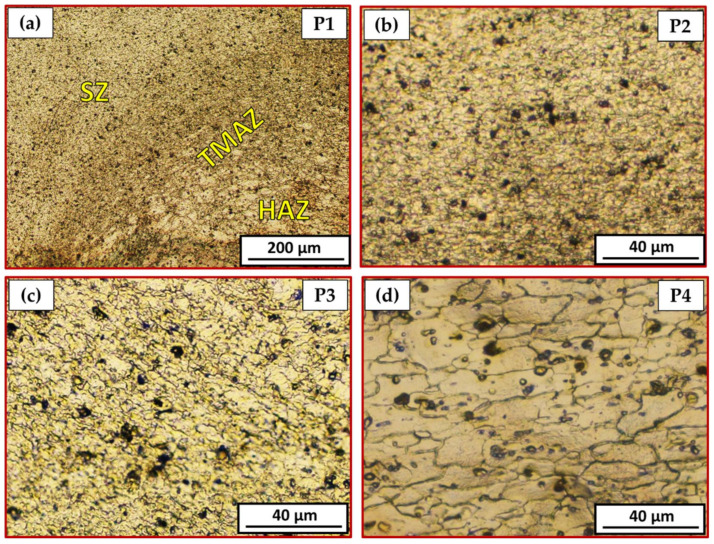
Different microstructure zones of the joint spot welded at 800 rpm where P1 is mixed zone of SZ/TMAZ/HAZ in (**a**), P2 is SZ in (**b**), P3 is TMAZ in (**c**), and P4 is HAZ in (**d**).

**Figure 14:**
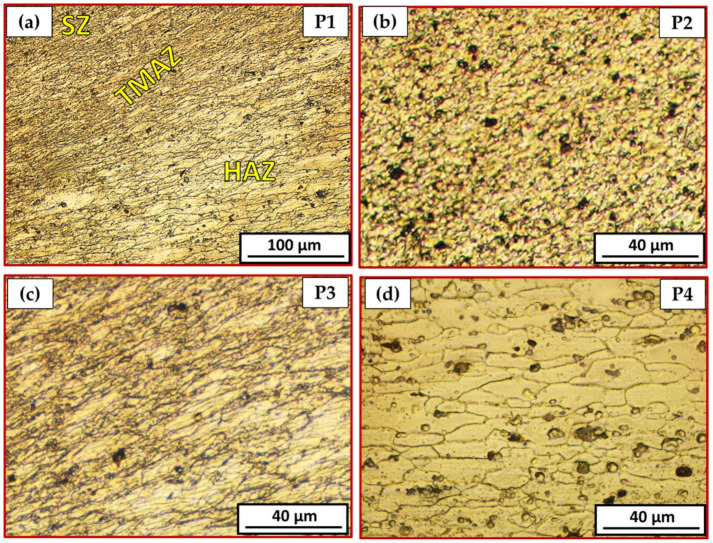
Different microstructure zones of the joint spot welded at 800 rpm where P1 is mixed zone of SZ/TMAZ/HAZ in (**a**), P2 is SZ in (**b**), P3 is TMAZ in (**c**), and P4 is HAZ in (**d**).

**Figure 15:**
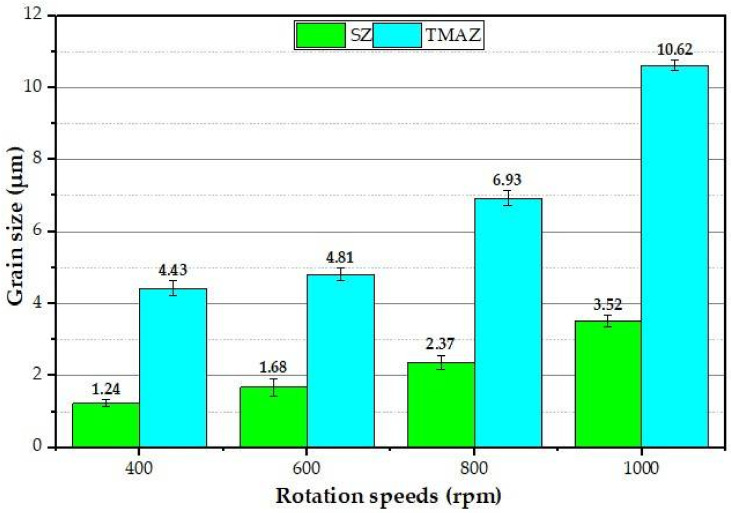
The mean grain size of the SZ and TMAZ of the joints spot welded at 400, 600, 800, and 1000 rpm rotation speed, and 3 s dwell time.

Figure 10 is a representative drawing example for the cross-section of the FSSWed joints to illustrate the locations (P1–P4) examined using an optical microscope to clarify the different zones achieved by the FSSW process at the various parameters. The optical micrographs in Figure 11a, Figure 12a, Figure 13a and Figure 14a illustrate various FSSWed zones (SZ, TMAZ, and HAZ) yielded after the FSSW at a constant dwell time of 3 s using the different rotation speeds of 400, 600, 800, and 1000 rpm. In addition, a keyhole is formed in the center of the FSSW lap joints. The microstructure analysis of the BMs (Figure 9) and S.Z regions confirm distinct modifications in grain morphologies and sizes (Figure 11b, Figure 12b, Figure 13b and Figure 14b). The elongated grains of the AA6082-T6 BM sheets are refined to equiaxed grains in a narrow range distribution at all the applied parameters. This change in the morphology and size of the grains is ascribed to dynamic recrystallization through the SZ [15,26,27,35]. The high temperature experienced and the amount of strain at this high temperature allows the dynamic recrystallization to take place [30,38]. It has been reported that the FSW of aluminum resulted in a high temperature and high strain rate that cause the formation of new fine equiaxed grain structure through dynamic recrystallization [39,40]. During the FSSW of aluminum, as noticed above, the temperature experience ranges between 220 to 350 °C, which is high enough to allow dynamic recrystallization at the high strain rate experienced. Thus, the grain size of the 2 mm AA6082-T6 BM reduced from 14.14 ± 1.5 µm to 1.24, 1.68, 2.37, and 3.52 µm for the stir zones of the joints spot welded at 400, 600, 800, and 1000 rpm, respectively, Figure 15. The average grain size of the SZ is increased with increasing the rotation speed from 400 to 1000 due to the increase in heat input (Figure 6). The coarse particles of (Fe, Mn)_3_SiAl_l2_ in the BMs have fragmented into small particles and redistributed in the aluminum matrix due to the stirring action in the SZ, whereas the fine precipitates (Mg_2_Si) are coarse in the SZ, which may be attributed to the dissolution and regrowth during the thermal cycle of welding, followed by air cooling [27,41]. Furthermore, the TMAZ grains (Figure 11c, Figure 12c, Figure 13c and Figure 14c) are noticeably rotated and deformed along with the material flow during the stirring action of the tool material. The grain size in the HAZ is affected by the frictional heat generation, not by plastic deformation (as shown in Figure 11d, Figure 12d, Figure 13d and Figure 14d). Thus, it is expected that a grain growth in HAZ compared to the BM grain size will be observed. The thermal exposure in the HAZ and the TMAZ during the FSSW process resulted in a coarsening of the precipitates. Finally, it can be concluded that the grain size of the different spot welding zones (SZ, TMAZ, HAZ) is directly related to the rotation speed when the other process parameters constant are kept constant. The mean grain size of the three zones increases with increasing tool rotation speed from 400 to 1000 rpm, as given in Figure 15.

### 3.5. Hardness Characterization

**Figure 16:**
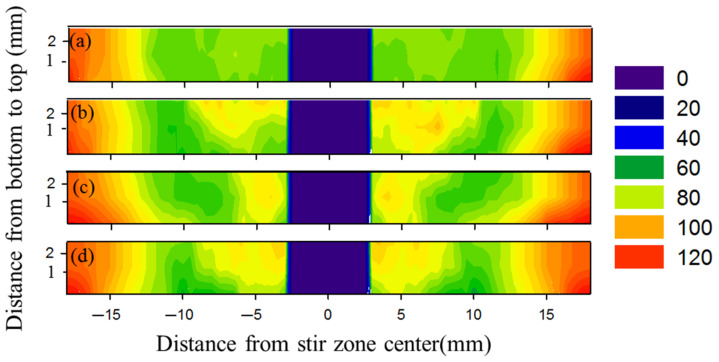
Two-dimensional hardness map of FSSW lap joints (**a**) 400, (**b**) 600, (**c**) 800, and (**d**) 1000 rpm.

Hardness was measured on the cross-sections of all the FSSWed joints produced at the rotational speeds of 400, 600, 800, and 1000 rpm and represented in color contoured maps, as shown in Figure 16. It is well known that the hardness through the thickness of the weld zones is controlled by thermal exposure during the FSW process [30]. Moreover, the state of the starting material affects the hardness behavior after FSW. For example, in this study, the starting material is T6 temper condition, which means fully hard through age hardening. Thus, the high thermal exposure is expected to result in hardness reduction in the weld region either due to the coarsening or the dissolution of the hardening precipitates [16,30,40,42,43,44,45]. In this study, the hardness contour map across the transverse cross section of the weld zone gives good representation of the hardness behavior. The AA6082-T6 BMs showed the mean hardness value of 127 ± 3 and 111 ± 2 HV_0.5_ for the 1 mm and 2 mm sheet thickness, respectively. Compared to the BM, the hardness of the weld zone of the FSSW joints significantly decreased at all the applied rotation speeds (Figure 16) due to annealing of the AA6082-T6 BMs caused by the frictional heat generated during the FSSW [5,27] and the FSW [8] processes. For each spot-welded joint among the weld zone, the minimum hardness values were observed in the HAZ due to the grain structure and overaging effects. In contrast the higher hardness was recorded in the SZ mainly due to the dynamic recrystallized equiaxed fine grain structure and the reprecipitation process that might take place upon the cooling cycle [30]. The hardness of the TMAZ shows lower values than the SZ and higher values of hardness than the HAZ. The increase of the TMAZ hardness over the HAZ may be ascribed to the high dislocation density promoted by the plastic deformation during the FSSW process [46,47,48]. The highest hardness values were 95 ± 2, 87 ± 3, and 80 ± 5 HV_0.5_ for the SZ, TMAZ, and HAZ, respectively, of the spot joint welded at 600 rpm (Figure 16b).

### 3.6. Tensile Shear Testing and Fracture Behavior

**Figure 17:**
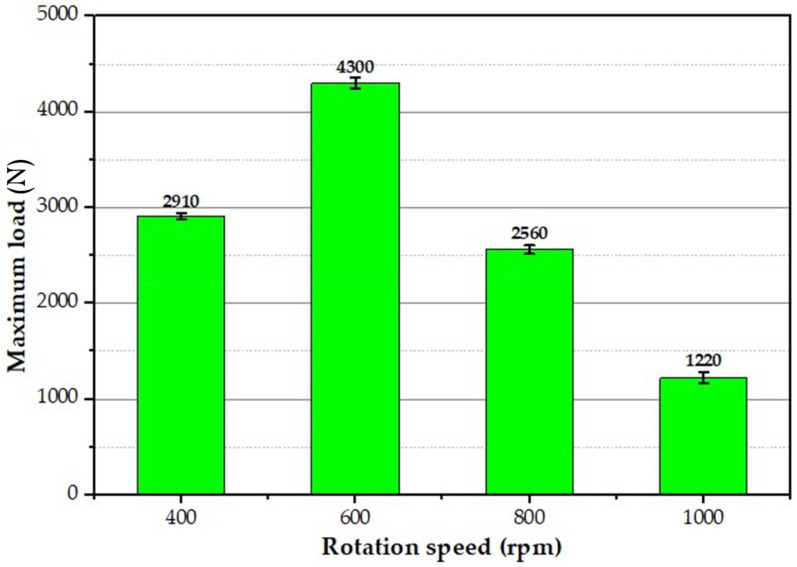
Maximum **t**ensile-shear load of the FSSWed joints processed at a constant dwell time of 3 s and different rotation speeds of 400, 600, 800, and 1000 rpm.

**Figure 18:**
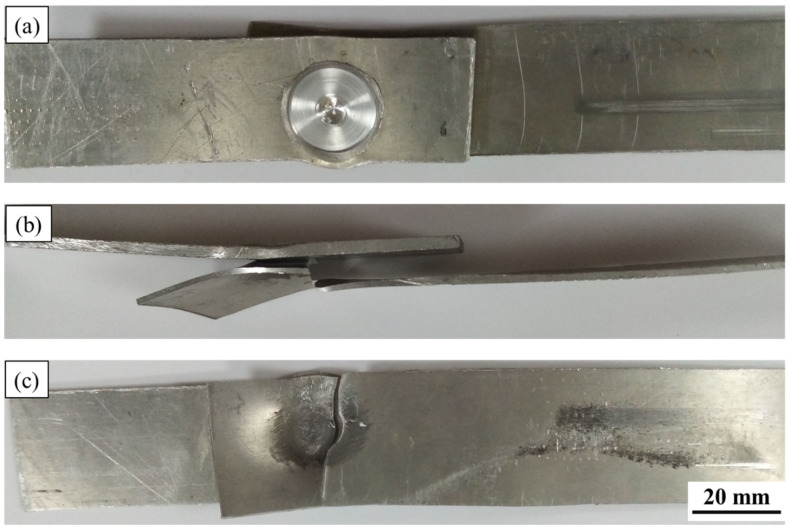
(**a**) top view, (**b**) side view, and (**c**) bottom view photographs of the fracture surface appearance of the FSSWed lap joint processed at 600 rpm and 3 s.

**Figure 19:**
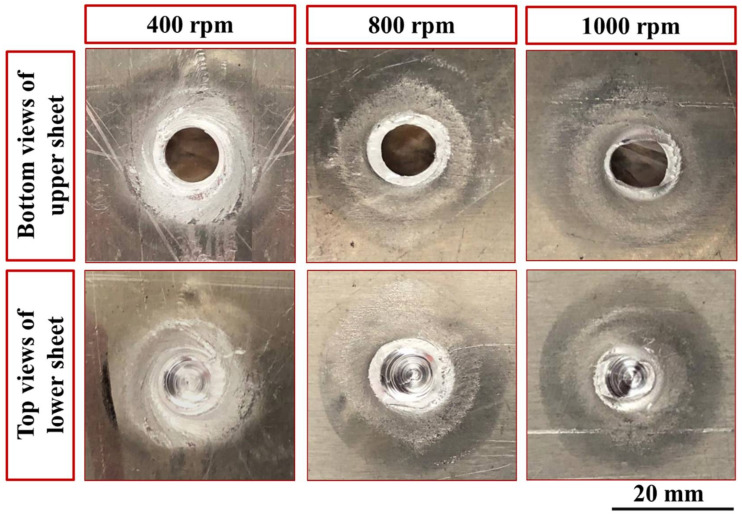
Photographs of fracture surface appearance of the FSSW lap joints processed at 400, 800, and 1000 rpm and 3 s dwell time.

Vehicle designers consider the tensile shear performance of the spot-welded joints while developing new models of vehicles. Thus, the tensile shear test for all the produced FSSWed joints was evaluated. It was reported that the tensile shear performance of the FSSWed was significantly affected by the welding process parameters [27,32,35]. The maximum tensile shear load of the FSSWed lap joints produced at the rotation speeds of 400, 600, 800, and 1000 rpm is illustrated in Figure 17. It can be seen here that the FSSW condition of 600 rpm rotation speed at 3 s dwell time shows the highest tensile shear load (4300 ± 30 N) compared to the load-carrying capacity of the other spot-welded joints. The two sheets of this spot lap joint are still connected after the tensile shear test, indicating high joint efficiency (Figure 18). This increase in tensile shear lap carrying capacity can be attributed to a larger fully bonded section size and lower hook height. In addition, the hardness improvement in the SZ is more than the hardness measured in the SZ of the other FSSWed joints. In contrast, the welded lap sheets produced at 400, 800, and 1000 rpm are completely separated during the tensile shear testing (Figure 19). The failed spot lap joint processed at 400 rpm may be ascribed to insufficient heat input, which affects in mixing during the welding of the dissimilar thickness AA6082-T6 lap joint. The tensile shear loads’ carrying capacity of the spot welds processed at higher rotation speeds of 800 and 1000 rpm show a decrease in the maximum tensile shear load. This drop in the tensile shear is likely due to the increased thermal softening in the SZ with rising heat input and lower thickness of the upper sheet underneath the shoulder. Xie et al. [49] and Ohashi et al. [50] concluded that the mechanical properties of the FSSWed lap joints are mainly governed by both the upper sheet underneath the shoulder and the bonded area. It should also be mentioned that the precipitate-free zones around the grain boundaries due to precipitate coarsening might have reduced the strength of SZ.

**Figure 20:**
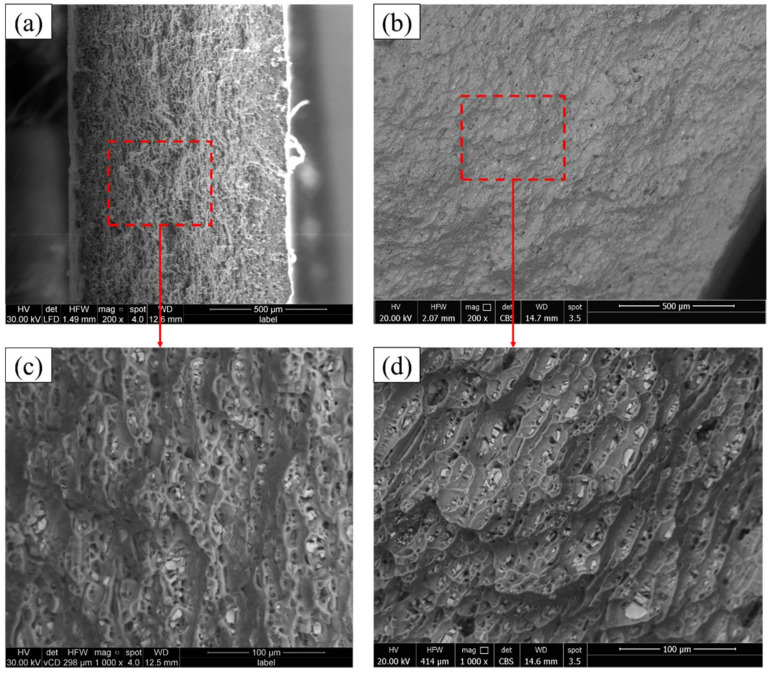
SEM images showing the fracture surface of AA6082-T6 BMs; (**a**,**c**) 1 mm and (**b**,**d**) 2 mm at different magnifications.

**Figure 21:**
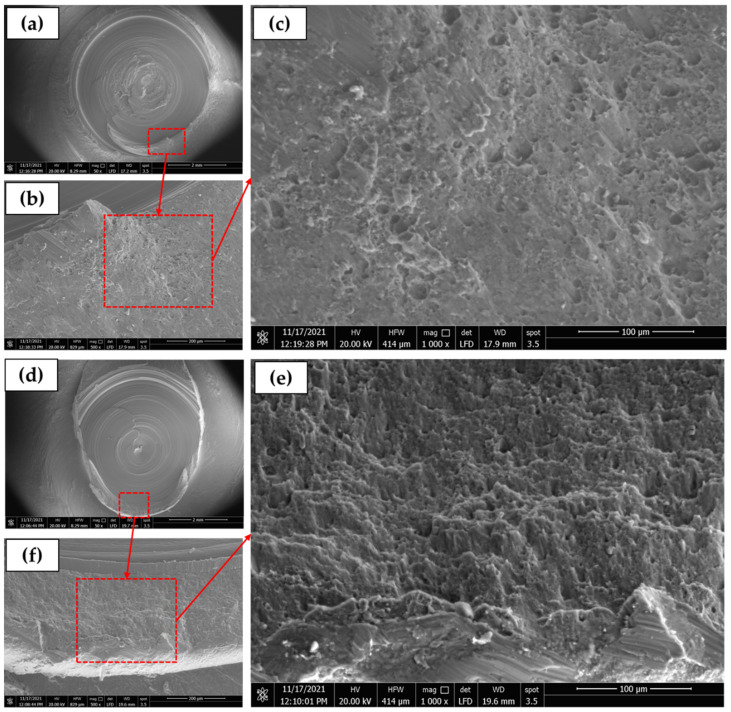
SEM images of FSSW lap joints at (**a**,**b**,**c**) 400 rpm and (**d**,**e**,**f**) 1000 rpm at different magnifications.

The fracture surfaces of the tensile-tested BMs are given in Figure 20. It can be seen here that the two fracture modes (ductile and brittle) are detected for the two sheet thicknesses of 1 mm (Figure 20a,c) and 2 mm (Figure 20b,d), that the ductile fracture showed by the aluminum matrix in terms of different dimple sizes in 1 mm sheet and shallow elongated dimples of 2 mm sheet thickness, while the brittle fracture was observed by the presence of precipitates of Mg_2_Si and (Fe, Mn)_3_SiAl_l2_. Microvoids due to the precipitates pullout are also detected on the fracture surface of the two sheets. The observed fracture surface is typically related to the microstructure features of AA6082-T6 BMs. Figure 21 illustrates the SEM fractography of the lower sheet of the FSSWed joint processed at a constant dwell time of 3 s and different rotation speeds of 400 (Figure 20a–c) and 1000 rpm (Figure 20d–f). The two spot lap joints were failed by the tensile shear mechanism. Under the tensile shear loading conditions, the microcracks begin at the partially bonded region at the tip of the hook and propagate preferentially in the horizontal direction at the spot joint interface, shearing the SZ causing failure. The fracture surface of the two joints at the lower sheets shows typically ductile features in terms of very small deep dimples compared to the BMs, indicating the grain refining of the SZ during the FSSW.

## 4. Conclusions

In the study, different AA6082-T6 thickness sheets of 1 mm and 2 mm were friction stir spot welded at a constant dwell time of 3 s and different rotation speeds of 400, 600, 800, and 1000 rpm. The spot-welded joints were characterized in terms of macro-, microstructure, hardness, and tensile shear testing and fracture surface. Based on the obtained results, the following conclusions can be outlined:

1. The applied FSSW parameters in terms of a constant dwell time of 3 s and different rotation speeds of 400, 600, 800, and 1000 rpm succeeded in welding two different thin sheet thicknesses of AA6082-T6 in spot lap joint configuration.
2. Among the produced joints, the spot-welded joint processed at 600 rpm achieved the highest SZ hardness of 95 ± 2 HV_0.5_ and maximum tensile shear load of 4300 ± 30 N.
3. The FSSW of AA6082-T6 temper condition significantly decreased the hardness in the weld zone (SZ, TMAZ, and HAZ) compared to the BMs, and the SZ showed higher hardness values than the TMAZ and HAZ.
4. The spot-welded joints processed at the lowest rotation speed of 400 rpm and those processed at the highest rotation speed of 1000 rpm failed in the SZ with a ductile fracture mode.

## Figures and Tables

**Table 1:** The chemical composition of aluminum alloy AA6082-T6.

Element	Mg	Mn	Si	Fe	Cr	Zn	Cu	Ti	Al
(wt. %)	0.6	0.4	0.75	0.5	0.2	0.2	0.1	0.1	Bal

**Table 2:** The mechanical properties of the two base material sheets of AA6082-T6.

AA6082-T6	Ultimate Tensile Strength(MPa)	Yield Stress(MPa)	Fracture Strength(MPa)	Hardness(HV)
1 mm sheet	301	296	236	127 ± 3
2 mm sheet	257	252	245	111 ± 2

**Table 3:** The maximum measured temperature during the FSSW process at different rotational speeds.

**Rotational Speeds (rpm)**	400	600	800	1000
**Peak Temperature (°C)**	236 ± 4	265 ± 3	308 ± 5	367 ± 3

## Data Availability

Data will be available upon request through the corresponding author.
